# Exosomes: Biological Carriers and Promising Tools for Cancer Immunotherapy

**DOI:** 10.3390/vaccines8030390

**Published:** 2020-07-16

**Authors:** Reem Saleh, Eyad Elkord

**Affiliations:** Cancer Research Center, Qatar Biomedical Research Institute (QBRI), Hamad Bin Khalifa University (HBKU), Qatar Foundation (QF), Doha 34110, Qatar; rsaleh@hbku.edu.qa

**Keywords:** cancer, exosomes, tumor microenvironment, immunosuppression, cancer immunotherapy

Exosomes are recognized as new therapeutic targets for cancer biomedicine and cancer immunotherapy [1]. More recently, emerging roles of exosomes as promising diagnostic tools and predictive biomarkers for cancer immunotherapy have been reported. Structurally, exosomes are small extracellular vesicles with 30 to 150 nm diameter, originate from one cell type (donor cell) and act as biological carriers [1]. They mediate cell-to-cell communication and exert biological activities by delivering their cargo contents, such as DNA, RNA, proteins or specific drugs, into target or recipient cells [2,3]. Exosomes are secreted from various cell types, including tumor and immune cells, and are capable of modifying the pathophysiological condition of recipient cells to convey messages which regulate distinct biological functions and transduce intercellular signaling [4]. 

Accumulating evidence suggests that exosomes can modulate anti-tumor immunity and play an important role in cancer progression. The cross-talk between tumor cells and immune cells is one of the core subjects which has received a great interest in the field of tumor immunology and has revolutionized the era of cancer immunotherapy. Exosome biogenesis, their role in immunoregulation and cell-to-cell communication, their future utilization in cancer immunotherapy and their potential role as predictive biomarkers for therapy responses were comprehensively reviewed by Jella et al. [5]. Authors have reviewed different methods for isolating and purifying exosomes from plasma samples, highlighting the advantages and disadvantages of each method [5]. These methods include ultracentrifugation, density gradient centrifugation, ultrafiltration, an immunoaffinity-based method and a polyethylene glycol (PEG)-based isolation method. Exomes can also be isolated from other biological fluids, such as seminal fluid, cerebrospinal fluid, serum and urine [6]. The methodologies for exosome isolation and purification vary, and can be selected upon the nature and physical properties of the biological fluid. The methodologies for characterizing purified exosomes to determine molecular characteristics, functional properties, biochemical composition, protein surface expression and morphology also differ across samples. These include flow cytometric analysis, Western blotting, the NanoSight method, transmission electron microscopy, dynamic light scattering, chromatography-mass spectrometry and immunohistochemistry [5].

Exosome biogenesis occurs within the donor cell as they form buds, early endosomes that mature into late endosomes, resulting in the formation of multivesicular bodies (MVBs) carrying DNA, mRNA and non-coding RNA species or protein [5]. These MVBs fuse with the plasma membrane of the donor cell to release exosomes into the extracellular environment and communicate with target or recipient cells. Exosomes are delivered to recipient cells via receptor-mediated endocytosis, where their content is secreted to exert a biological function. The biological function of the exosome is dictated by the type of cargo content and the type of recipient and donor cells. Exosomes can trigger signaling pathways which favor tumor growth/metastasis/angiogenesis, and promote tumor escape from immune recognition and resistance to therapy [5]. Within the tumor microenvironment (TME), tumor cells can release exosomes, called tumor-derived exosomes, containing growth factors (e.g., TGF-β) and microRNAs (e.g., miR-423-5p and miR-675), which favor epithelial–mesenchymal transition, tumor growth/metastasis and angiogenesis [7,8,9]. Tumor-derived exosomes can also trigger the induction and expansion of myeloid-derived suppressor cells (MDSCs), and reprogram fibroblasts within the tumor tissue to be converted into cancer-associated fibroblasts (CAFs) with pro-tumorigenic and immunosuppressive properties [10,11,12]. Moreover, tumor-derived exosomes can act on dendritic cells (DCs) within the TME and impair their function to become tolerogenic DCs, thereby inhibiting the activation of natural killer (NK) cells, cytotoxic CD8^+^ T cells and CD4^+^ effector T cells, and favoring the activation of T regulatory cells (Tregs), immunosuppressive subsets of T cells which support tumor growth and metastasis [13,14]. Another mechanism by which tumor-derived exosomes are capable of triggering immune escape is via the induction of tumor antigen loss, thereby rendering the activation of tumor-specific CD8^+^ T cells and NK cells [15]. Other reports showed that tumor-derived exosomes can reduce the number of CD8^+^ T cells either via diminishing their proliferation by reducing the levels of IL-2, or inducing their apoptosis via FasL-TRAIL pathway or galectin 9 [16,17]. On the other hand, studies have shown the involvement of tumor-derived exosomes in the activation and expansion of Tregs, perhaps via the release of TGF-β [15]. Moreover, there is some evidence demonstrating the role of tumor-derived exomes in triggering resistance to chemotherapeutic drugs either by effluxing the drug from tumor cells or initiating the signaling of multidrug resistance, thereby reducing tumor cell sensitivity to therapies [18,19]. 

Jella et al. have also described the potential benefits of utilizing exosomes as a therapeutic approach to aid cancer immunotherapy in inducing potent long-lasting immune responses against tumor cells and alleviating tumor-mediated therapy resistance [5]. The authors have reviewed the advantages of using exosomes over other nanoparticles, which make them a promising approach for cancer immunotherapy; these advantages include that exosomes have a long circulating half-life, high specificity for target cells and reduced risks of toxicity. In addition, evidence from preclinical models and in vitro studies were provided to highlight the benefits of exosomes, particularly DC-derived exosomes, in promoting anti-tumor immune responses and suppressing tumor growth [5]. In light of the encouraging results obtained from preclinical models and in vitro studies, the safety and efficacy of exosomes were evaluated in clinical trials. A report from a phase I clinical trial demonstrated the safety, but not the efficacy, of autologous DC-derived exosomes loaded with MHC II and tumor MAGE peptides in metastatic melanoma patients [20]. A phase I clinical trial in colorectal cancer patients evaluated the combined use of GM-CSF treatment and ascites-derived exosomes, and showed the ability of combined therapy in inducing tumor-specific cytotoxic T cell-mediated responses [21]. Another phase I clinical trial demonstrated the safety and therapeutic efficacy of autologous DC-derived exosomes pulsed with tumor MAGE peptides in improving anti-tumor immunity and extending disease stability in patients with advanced non-small cell lung cancer (NSCLC) [22]. In a phase II clinical trial, the administration of exosomes loaded with IFN-γ and MHC class I and II in patients with advanced NSCLC boosted NK cell-mediated anti-tumor immune response, and extended overall survival and progression-free survival in 50% of the participating patients [23]. Moreover, there is evidence suggesting the benefits of using chimeric antigen (CAR) T cell-derived exosomes in controlling the immune related adverse event, cytokine storm syndrome, which is induced by CAR T cell therapy in cancer patients, and in improving the specificity of therapy [24]. Hence, exosomes have the potential of improving the clinical response to CAR T cell therapy to be more tolerable and more effective. Currently, there are no ongoing or registered phase III clinical trials for exosome-based cancer therapy, and all of them are phase I or II trials [25,26].

Apart from being a potential immunotherapeutic tool, exosomes have the potential to serve as non-invasive liquid biomarkers in many cancer patients to indicate disease activity or tumor stage [27,28,29,30]. A study by Chen et al. showed that the level of circulating PD-L1 exosomes is high in metastatic melanoma patients; this level varied during the course of anti-PD-1 therapy, suggesting the possibility of utilizing PD-L1 exosomes as a predictive biomarker for the response of patients to anti-PD-1 therapy and distinguish responders from non-responders [31]. Results from a phase I clinical trial showed that tumor-derived exosomes and T cell-derived exosomes in patients with head and neck squamous cell carcinoma (HNSCC) can predict the clinical response to a combined therapy of cetuximab, ipilimumab and radiation [32]. However, Jella et al. Ref. [5] did not discuss the potential adverse effects of using exosomes in cancer therapy as they could weaken the anti-tumor immune response by sequestering tumor-reactive antibodies [33]. In vitro and in vivo evidences have suggested that human epidermal growth factor receptor-2 positive (HER2^+^) breast tumor-derived exosomes can reduce the sensitivity of tumor cells to trastuzumab-based therapy by inhibiting the activity of the drug and promote HER2^+^ tumor aggressiveness [33].

In conclusion, exosomes can vary in terms of their cellular origin, and molecular and functional characteristics, and have different specificities based on their cellular source. Although exosome-mediated effects can be specific to tumor sites, and can effectively inhibit the tumor progression and enhance anti-tumor immunity, there are potential risks of adverse immune events [3]. Additionally, current challenges associated with exosome isolation/purification need to be overcome to increase the efficiency of exosome isolation, and increase their yield and purity [34]. Future research should focus on developing more standardized methods and suitable protocols for exosome isolation and purification, depending on the nature of the biological fluid. It should also focus on improving the methods for characterizing purified exosomes to increase the efficiency of exosomes in exerting biological functions with a high specificity and to promote their application in clinical studies. Moreover, it is imperative to understand how to select appropriate loaded exosomes for different tumor types, and how to deliver exosomes to recipient cells accurately and efficiently to improve current approaches of exosome application as immunotherapeutic agents. Further investigations are also required to assess the utilization of exosomes as biomarkers to predict clinical responses of cancer patients to a particular therapy.
